# Undiagnosed hypertrophic obstructive cardiomyopathy during transcatheter aortic valve replacement: a case report

**DOI:** 10.1186/s13256-018-1904-8

**Published:** 2018-12-18

**Authors:** Kevin R. Olsen, Joseph E. LaGrew, Caleb A. Awoniyi, J. Christopher Goldstein

**Affiliations:** 1 Department of Anesthesiology, University of Florida College of Medicine, Gainesville, Florida USA; 2 North Florida/South Georgia Veterans Affairs Medical Center, 1601 SW Archer Road, Gainesville, Florida 32608 USA

**Keywords:** Aortic stenosis, TAVR, Transcatheter aortic valve replacement, Hypertrophic obstructive cardiomyopathy

## Abstract

**Background:**

Transcatheter aortic valve replacement is indicated for severe symptomatic aortic stenosis in patients who have a very high or prohibitive surgical risk as assessed pre-procedurally by the Society of Thoracic Surgery Risk Score, EuroSCORE (II), frailty testing, and other predictors. When combined with another left ventricular outflow tract obstruction, careful consideration must be taken prior to proceeding with transcatheter aortic valve replacement because an additional masked left ventricular outflow tract pathology can lead to challenging hemodynamics in the peri-deployment phase, as reported in this case.

**Case presentation:**

A 56-year-old Caucasian man with multiple comorbidities and severe aortic stenosis underwent transcatheter aortic valve replacement under monitored anesthesia care. During the deployment phase, he developed dyspnea that progressed to pulmonary edema requiring emergent conversion to general anesthesia, orotracheal intubation, acute respiratory distress syndrome-type ventilation, and vasopressor medications. Intraoperative transesophageal echocardiography was performed and hypertrophic obstructive cardiomyopathy with systolic anterior motion of the mitral valve was discovered as an underlying pathology, undetected on preoperative imaging. After treatment with beta blockers, fluid resuscitation, and alpha-1 agonists, he stabilized and was eventually discharged from our hospital without any lasting sequelae.

**Conclusions:**

Patients with aortic stenosis most often develop symmetric hypertrophy; however, a small subset has asymmetric septal hypertrophy leading to left ventricular outflow tract obstruction. In cases of severe aortic stenosis, however, evidence of left ventricular outflow tract obstruction via both symptoms and echocardiographic findings may be minimized due to extremely high afterload on the left ventricle. Diagnosing a left ventricular outflow tract obstruction as the cause of hemodynamic instability during transcatheter aortic valve replacement, in the absence of abnormal findings on echocardiogram preoperatively, requires a high index of clinical suspicion. The management of acute onset left ventricular outflow tract obstruction intraoperatively consists primarily of medical therapy, including rate control, adequate volume resuscitation, and avoidance of inotropes. With persistently elevated gradients, interventional treatments may be considered.

## Background

Efforts at percutaneous valve replacement have been described in the literature as early as 1965 in an animal model [1]. Progress in developing this intervention was slow at first, with the first successful valve placement in a human 35 years later [2]. Soon after, the growth in percutaneous valve replacement accelerated. The first transcatheter aortic valve replacement (TAVR) was described by Cribier and colleagues in 2002 [3], and by 2010, it was estimated that over 30,000 valves had been implanted by this procedure [4]. With large, multicenter randomized controlled trials confirming its efficacy [5], consensus guidelines supported TAVR for patients with prohibitive risk (grade I recommendation) and for those at high risk (grade IIa recommendation) for surgical aortic valve replacement [6]. The most recent guidelines from 2017 now include patients at an intermediate risk [7].

As indications and experience with TAVR have expanded, so has knowledge of common associated risks. Short-term and intermediate follow-up have shown significant risk of mortality as well as major bleeding, myocardial infarction, cerebrovascular events, new atrial fibrillation or other conduction abnormalities, aortic regurgitation, acute kidney injury, vascular injury, coronary obstruction, and valve malpositioning [8]. Rarer associated adverse effects in this relatively new modality are still being elucidated. There have been few case reports of hypertrophic obstructive cardiomyopathy (HOCM) after TAVR [9, 10] despite its relatively common association with aortic stenosis (AS) [11].

This case describes an episode of acute hemodynamic instability immediately following valve deployment in TAVR from left ventricular outflow tract (LVOT) obstruction secondary to HOCM. The patient provided written consent for publication of this report.

## Case presentation

### Intraoperative course

**Fig. 1:**
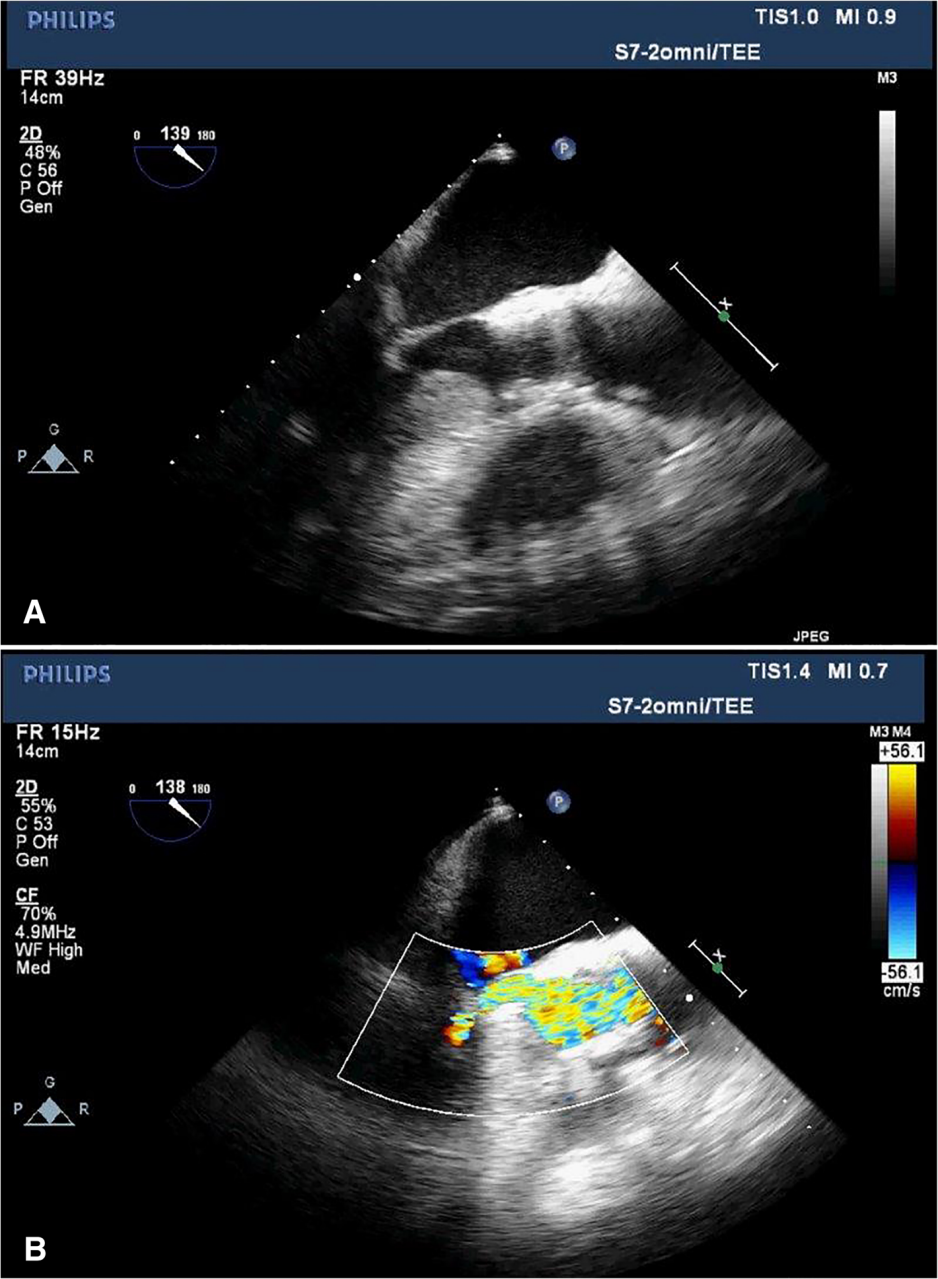
Intraoperative transesophageal echocardiogram, mid-esophageal long-axis view obtained after rapid pacing and valve deployment demonstrating (**a**) systolic anterior motion of the mitral valve leading to severe restriction of flow (**b**) as demonstrated on color Doppler imaging

**Fig. 2:**
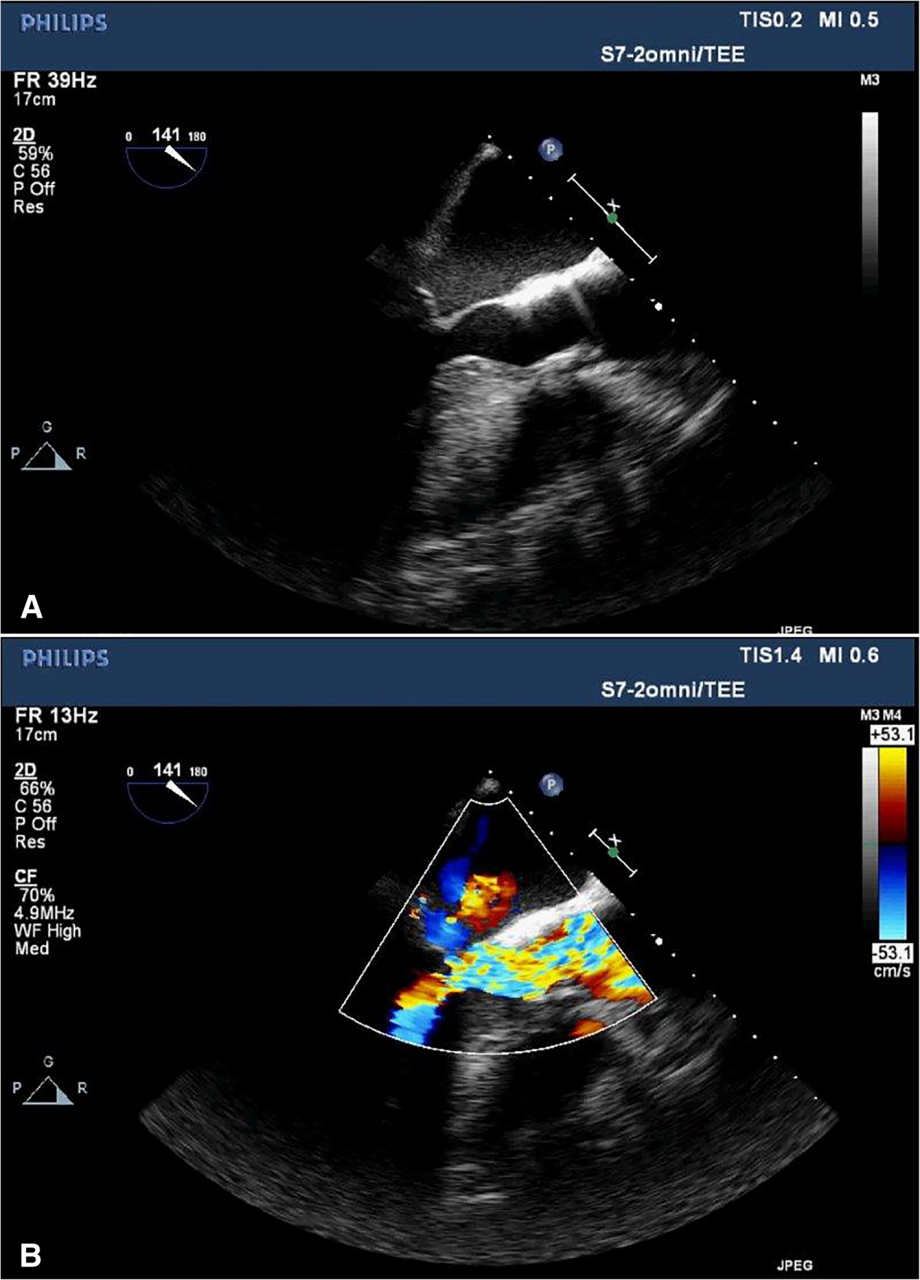
Intraoperative transesophageal echocardiogram, mid-esophageal long-axis view obtained after rate control and treatment with alpha-1 agonists demonstrating (**a**) improved left ventricular outflow tract diameter with no apparent systolic anterior motion and (**b**) improved subvalvular flow on color Doppler imaging

A 56-year-old Caucasian man with severe AS (valve area 0.81 cm^2^, mean gradient 54 mmHg), high Society of Thoracic Surgeons (STS) score (> 8), moderate chronic obstructive pulmonary disease with forced expiratory volume in 1 second (FEV_1_) of 1.9 L at 53% of predicted, Crohn’s disease (on immunosuppressive therapy), hypertension, and non-obstructive coronary artery disease presented for a TAVR procedure under monitored anesthesia care. An echocardiogram and subsequent left ventricular hemodynamic study completed as part of the preoperative evaluation showed symmetric, concentric left ventricular hypertrophy with no left ventricular outflow gradient. His early intraoperative course was unremarkable. Following successful deployment of the transcatheter valve and cessation of rapid ventricular pacing at 180 beats per minute, he became hypotensive, tachycardic, and short of breath. Despite escalating doses of phenylephrine, ephedrine, vasopressin, norepinephrine, and epinephrine, he remained profoundly hypotensive and unresponsive. He continued to decompensate and developed acute pulmonary edema, requiring oral suctioning and rapid sequence intubation. Given his deterioration immediately following rapid pacing and valve deployment with poor response to epinephrine, an LVOT obstruction was considered, but other etiologies were included in the differential (Table 1). Consequently, inotropic medications were ceased and rate-control and afterload-increasing medications (esmolol, phenylephrine) were prioritized with marked improvement in symptoms. An expedited intraoperative transesophageal echocardiography (TEE) assessment confirmed the presence of hypertrophic cardiomyopathy pathology causing LVOT obstruction with associated mitral valve systolic anterior motion, as shown by the “hockey sticking” of the anterior mitral valve leaflet into the LVOT in Fig. 1a. The physiologic consequence of this decompensation is clearly represented in Fig. 1b, showing a near obliteration of flow across the LVOT. The initial peak gradient across the LVOT obstruction reached 70 mmHg, and we considered performing an urgent septal alcohol ablation in the event that the gradient remained refractory to our attempts at medical management. Beta blockade with metoprolol was titrated for rate control, a phenylephrine infusion was started to maintain systemic vascular resistance, and fluid resuscitation to achieve euvolemia was guided by TEE. This resulted in a significant improvement of the LVOT obstruction (peak gradient of 25 mmHg, mean of 13 mmHg) as shown in Fig. 2a and b. Intubated, paralyzed, and showing stable vital signs, our patient was transported without external pacing to the intensive care unit. Subsequent arterial blood gas was normal with improving respiratory status.

**Table 1:** Considerations for decompensation after transcatheter aortic valve replacement include cardiac pathology, drug-related adverse effects, valve dysfunction, and pulmonary disease with diagnostic considerations based on suspected cause.

Acute Hemodynamic Decompensation during TAVR	
Differential	Diagnostic and Treatment Considerations
MI, CHF exacerbation, cardiac arrest, arrythmia, cardiac tamponade, acute cordae rupture, aortic dissection, annular rupture	Consider: 12-lead EKG, TTE, TEE
Anaphylaxis, vasodilating medications, inhaled anesthetics	Verify medications & allergies, assess depth of anesthesia
Valve migraton, valve embolization, valve malfunction	Consider: TEE, TTE, fluoroscopy
Pulmonary embolism, acute pulmonary edema, pneumothorax, hemothorax, hypoventilation, hypoxemia	Consider: 12-lead EKG, TTE, TEE, auscultation, CXR

*CHF* congestive heart failure, *CXR* chest X-ray, *EKG* electrocardiogram, *MI* myocardial infarction, *TEE* transesophageal echocardiography, *TTE* transthoracic echocardiography

### Postoperative course

He was mechanically ventilated, sedated, and remained on a phenylephrine infusion until postoperative day (POD) 1 when he became responsive. A transthoracic echocardiogram showed moderate to severe concentric left ventricular hypertrophy, normal left ventricle (LV) systolic function, ejection fraction of 60%, and a normally functioning prosthetic valve with peak gradient of 27 mmHg and mean systolic gradient of 10 mmHg. An overlying LVOT gradient of 40 mmHg that was late peaking was also observed. He was weaned from vasopressor medications and extubated on POD 2. He was ambulatory and able to move to the ward on POD 4, and was discharged on POD 5. Post-discharge, his LVOT gradient gradually increased again and his HOCM was deemed refractory to medical management. Eight months after his TAVR, he received an alcohol ablation which successfully relieved the LVOT obstruction.

## Discussion

This case highlights the importance of understanding the relationship between AS and HOCM. Most patients with AS develop symmetric hypertrophy; however, a small subset have asymmetric septal hypertrophy leading to LVOT obstruction. This is detectable on echocardiography by measuring the ratio of ventricular septum thickness relative to the posterior ventricular free wall (values greater than 1.3 are considered diagnostic). In cases of severe AS, however, LVOT obstruction may be minimized due to extremely high afterload on the LV. Clinical suspicion in the absence of abnormal findings on echocardiogram can be substantiated by a disproportionately sharp increase in stenosis (typical change in valve area is 0.1–0.19 cm^2^/year) [10] and confirmed with invasive hemodynamic evaluation. In the case presented here, cardiac catheterization demonstrated no septal asymmetry and no subvalvular gradient, the latter probably due the severity of this patient’s AS.

With the abrupt decrease in afterload after placement of the new valve in the hypertrophied LV of patients with AS, preexisting HOCM may worsen, or new dynamic intraventricular gradients (DIG) may develop [12]. These gradients are defined as a maximum flow velocity greater than 2.5 m/s, and can result in LVOT obstruction even in patients without previous subvalvular obstruction. Predictors of DIG include small LV end diastolic diameter, high ejection fractions, high intraventricular septum to posterior wall ratios, high valve gradients, and small LV masses [13]. Patients with new gradients are treated similarly to those with preexisting HOCM with improvement in their symptoms as hypertrophy decreases following aortic valve replacement. Although the only other case report of DIG following TAVR presented on POD 1 [9], LV mechanics have been shown to change early following valve replacement [14], and it is possible that the symptoms here resulted from a new onset of DIG. Our patient had only two risk factors, and on follow-up his symptoms and LVOT gradient worsened, making this diagnosis less likely. Other considerations for acute decompensation following valve deployment during TAVR include cardiac pathology, adverse response to medications, valve dysfunction, and pulmonary pathology (Table 1).

In the management of acute onset LVOT obstruction intraoperatively, medical therapy is the mainstay of treatment. Rate control, adequate volume resuscitation, and avoidance of inotropes are first-line interventions. With persistently elevated gradients, procedural interventions should be considered based on individual patients’ surgical risk. As candidates for TAVR now include those with intermediate surgical risk, myomectomy or urgent septal alcohol ablation may be considered. In this particular patient, myomectomy would probably carry a high morbidity and mortality. In addition, reseating the prosthetic valve inferiorly to sit over the bulging septum has been reported as a successful treatment of acute onset of subvalvular gradient [15].

The case presented here was most consistent with HOCM associated with AS (worsening course following correction of AS improved by septal alcohol ablation) but presented without some of the typical signs, such as asymmetric septal hypertrophy or LVOT gradient on invasive hemodynamic studies. It worsened acutely after TAVR and ultimately led to intraoperative left heart failure, flash pulmonary edema, and hemodynamic instability. Although previous reports demonstrated that LVOT obstruction can evolve subacutely following TAVR [9, 10], the acute decompensation demonstrated in this patient emphasizes the importance of understanding the relationship between these processes. Prompt diagnosis of LVOT obstruction following aortic valve deployment for the patient presented here prevented a potentially catastrophic outcome.

## Conclusions

HOCM associated with AS is an important pathophysiology to consider in the event of decompensation during TAVR. Unresponsiveness to inotropic agents along with intraoperative TEE findings can help identify this condition, but vigilance and a high degree of suspicion are important factors as well. Even a thorough preoperative workup may miss this potentially lethal pathophysiology. Intraoperative management consists of afterload-increasing agents (that is, phenylephrine), adequate preload (that is, fluid resuscitation), and maintaining sinus rhythm and avoiding tachycardia (that is, beta blockers). Patients decompensating in spite of maximal medical therapy may benefit from emergent alcohol ablation or myomectomy.

## Abbreviations

AS: Aortic stenosis
DIG: Dynamic intraventricular gradients
HOCM: Hypertrophic obstructive cardiomyopathy
LV: Left ventricle
LVOT: Left ventricular outflow tract
POD: Postoperative day
TAVR: Transcatheter aortic valve replacement
TEE: Transesophageal echocardiography
